# Use of Black-and-White Digital Filters to Optimize Visualization in Cataract Surgery

**DOI:** 10.3390/jcm11144056

**Published:** 2022-07-13

**Authors:** Otman Sandali, Joutei Hassani Rachid Tahiri, Ashraf Armia Balamoun, Cedric Duliere, Mohamed El Sanharawi, Vincent Borderie

**Affiliations:** 1 Centre Hospitalier National d’Ophtalmologie des XV-XX, Research Team 968, Institut de la Vision, Pierre & Marie Curie University Paris 06, 75012 Paris, France; vincent.borderie@upmc.fr; 2 Service de Chirurgie Ambulatoire, Hôpital Guillaume-de-Varye, 18230 Bourges, France; 3 Service de Chirurgie Ambulatoire, Centre Hospitalier de Granville, 50400 Granville, France; tjhr78@hotmail.com; 4 Watany Eye Hospital (WEH), Cairo 11775, Egypt; ashrafarmia@gmail.com; 5 Watany Research and Development Centre, Cairo 11775, Egypt; 6 Ashraf Armia Eye Clinic, Giza 12655, Egypt; 7 Hôpital Lariboisiere AP-HP, 75010 Paris, France; cedricduliere@gmail.com; 8 Service d’Ophtalmologie, Centre Hospitalier de Châteaudun, 28200 Châteaudun, France; mohamed.elsanharawi@gmail.com

**Keywords:** cataract surgery, heads up three-dimensional (3D) digital visualization system, black-and-white (BW) filter, contrast, visualization, color channels

## Abstract

Purpose: To evaluate the effect of a black-and-white (BW) filter on the optimization of visualization at each stage of cataract surgery. Methods: Prospective, single-center, single-surgeon, consecutive case series of 40 patients undergoing cataract surgery with BW filter. Surgical images and videos were recorded with and without the BW filter at each stage of cataract surgery. Contrast measurements of surgical images and subjective analysis of video sequences were performed. Results: The surgeons assessed the BW filter to optimize the tissue visibility of capsulorhexis contours, hydrodissection fluid wave perception, the contrast of instruments through a nucleus during phaco-chop, and subincisional cortex contrast through the corneal edema. Despite the higher contrasts’ value obtained with BW filter images during nucleus removal, posterior capsular polishing and viscous removal, the surgeons subjectively reported no significant advantage of using a BW filter. Standard color images were found to be better for localizing the limbal area during incision and for nucleus sculpture to assess groove depth. Conclusions: In conclusion, we describe here the potential indications for BW filter use at particular stages in cataract surgery. A BW filter could be used, with caution, in cases of poor visualization.

## 1. Introduction

Cataract surgery is one of the most frequently performed surgical interventions worldwide [1]. The development of phacoemulsification technology, instrumentation and surgical techniques has improved both the efficacy and safety of procedures [2].

A three-dimensional (3D) heads-up system was recently introduced for both vitreoretinal and cataract surgery, radically improving the quality of surgical visualization. This system has several advantages over conventional microscopes, including digitally enhanced imaging, which provides high-quality visualization, with lower levels of illumination during surgery, increasing patient comfort [3,4]. The 3D system extends the depth of field, thereby improving visualization of the anterior chamber at high magnification. This makes intraocular interventions safer and helps to minimize postoperative corneal edema in eyes with a shallow anterior chamber [5]. The ocular-free design of the 3D system not only improves ergonomic conditions for the surgeon, but also makes it possible to perform surgery in unusual and challenging positions, by rotating the microscope for cataract surgery in patients who are unable to remain supine, or through extending the peripheral retinal view in retinal surgery [6,7].

Another interesting feature of this system is that the digital camera of the 3D system allows the use of color filters, which can be modified during surgery, creating new possibilities for improving tissue visualization.

Black-and-white (BW) filters have classically been used to enhance medical image quality and tissue contrast [8]. The use of BW filters was recently reported to facilitate the visualization of internal limiting membrane peeling rhexis in vitreoretinal procedures in patients with retinal pigment epithelium atrophy [9].

Good visualization during surgery is essential to ensure the safety of procedures. There are certain conditions such as corneal opacities, corneal edema, and poor red reflex perception that can decrease the quality of visualization.

The aim of this study was to evaluate the effect of a black-and-white filter on the optimization of visualization at each stage in cataract surgery, in a prospective series.

## 2. Materials and Methods

We prospectively studied the first 40 patients scheduled to undergo phacoemulsification surgery at Guillaume de Varye Hospital (Bourges, France) between January and February 2022. This study was approved by the ethics committee of our institution, and informed consent was obtained from the patients before inclusion. The study was performed in accordance with the Declaration of Helsinki. The patients included in this study had grade two to three cataracts, according to a simplified nuclear classification score based on posterior nuclear color [10].

### 2.1. Technique

All interventions were performed by the same experienced surgeon (O.S.) using a 3D digital visualization system (NGENUITY^®^, Alcon, Fort Worth, TX, USA) in which a high-dynamic range video camera was connected to the microscope (Lumera 700 Carl Zeiss Meditec, Jena, Germany) and replaced the oculars. The color temperature of the halogen lamp used was 3400 Kelvin. The light intensity on the OPMI Lumera 700 microscope was set to 40% of the maximum value. The iris diaphragm of the digital video camera was set to approximately 30% open for 3D surgery.

All of the operations were performed under topical anesthesia. The Constellation (Alcon Surgical, Ft. Worth, TX, USA) microsurgical system was used for ultrasound phacoemulsification, with a 45-degree Kelman 0.9 mm mini-flared TurboSonics tip. A 2.2 mm clear superior corneal incision was made and a capsulorhexis of about 5.0 mm in diameter was created with forceps. A cortical cleaving hydrodissection was then performed. The nucleus was emulsified by the in-the-bag phaco-chop technique or the sculpture technique, depending on the surgeon’s assessment of nuclear hardness before phacoemulsification. The phaco-chop technique was, thus, used for hard cataracts, whereas the sculpture technique was performed for soft cataracts.

The BW filter was applied before each surgery, by decreasing the saturation of the digital camera from 90% (standard color) to 0. The surgeon systematically removed this filter when he considered the visual quality to be insufficient, at any time during surgery.

The other image parameters remained unchanged, with settings of 47.80 for brightness, 54.90 for contrast, 1.20 for gamma, and two for hue. The cyan-red, magenta-green, and yellow-blue color filters of the Ngenuity camera were maintained in a neutral position and were set to 100.

The images were captured with and without the BW filter of the 3D system at precisely the same moments, in each stage of the cataract surgery: before the incision, during the capsulorhexis, nucleus sculpture, fragment removal, cortex aspiration, posterior capsule polishing, and viscous removal.

For the comparison of other surgical sequences when it was not possible to interrupt the procedure to capture images with both the color and BW channels, such as the hydrodissection, and phaco-chop, all the procedures were recorded with the BW filter of the NGENUITY camera and without the filter with the Zeiss recorder of the microscope. Comparability was ensured by adjusting the images to the same resolution: 1920 × 1080 pixels.

### 2.2. Data Collection

**Figure 1:**
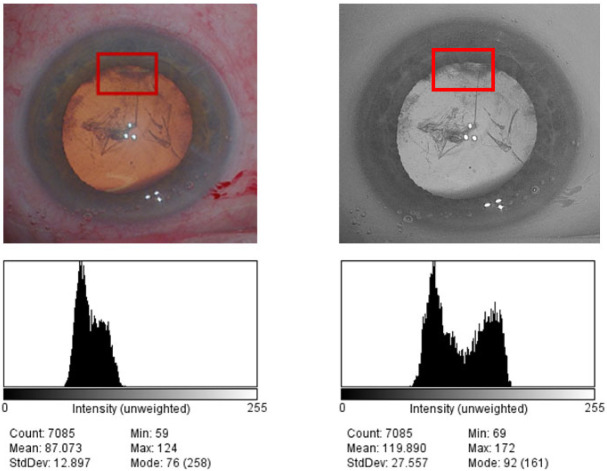
Standard color and BW filter images obtained during capsulorhexis and their luminance histograms in pixel values using image J software. The contrast of the region of interest which represents the difference in the standard deviation (DSD) of pixel intensity values was 12,897 for standard colors and 27,557 for BW filter.

We used ImageJ (version 1.52a, U.S. National Institutes of Health (NIH), Bethesda, MD, USA) to draw the regions of interest for the analysis. The contrast was used to quantify the variation of pixel intensity, which was measured as the difference in the standard deviation (DSD) of pixel intensity values (Figure 1).

For the assessments of the contribution of the BW filter in challenging conditions, only the areas in which visualization quality was poor were images selected for contrast analysis.

The images and videos that were recorded with and without the BW filter were analyzed by two experienced surgeons (OS, ME), who rated the quality of tissue contrast visualization at each stage of surgery with a scale (one to three; one = worse, two = no significant difference, three = better). The visualization quality was assessed for limbal identification before the corneal incision, capsulorhexis contour visibility, hydrodissection fluid wave perception, instrument visibility through the nucleus during phaco-chop, nucleus groove during sculpting, nucleus removal, subincisional cortex removal, residual cortical debris during posterior capsular polishing, and viscous in the anterior chamber at the end of a surgery.

### 2.3. Statistical Analysis

The results were presented as mean ± standard deviation for the continuous variable and as proportions (%) for categorical variables. Paired *t*-test and non-parametric Wilcoxon signed rank test were used for statistical comparisons between black/white and colors contrast in different stages of the cataract surgery.

Regarding the analysis and comparison of the black/white and color photos, agreement between observers was assessed using a chi-square test and the κ statistic, the latter allowing for the estimation of the level of agreement while accounting for the agreement obtained by chance. The k statistics were interpreted using the ranges that were suggested by Landis and Koch: 0 to 0.20, slight agreement; 0.21 to 0.40, fair agreement; 0.41 to 0.60, moderate agreement; 0.61 to 0.80, substantial agreement; and more than 0.80, almost perfect agreement. Any instances of *p* < 0.05 were considered statistically significant. The statistical analysis was carried out using SPSS for Windows version 27.0 (SPSS, Inc., Chicago, IL, USA).

## 3. Results

We included 40 eyes from 40 patients in this study. The surgeries were performed using the phaco-chop technique on 26 patients’ eyes and the sculpture technique for the remaining 14 patients’ eyes. No ocular complication occurred in any of the interventions. There was good agreement between the surgeon observers who were assessing for visualization quality (O.S, E.M) (Table 1).

### 3.1. Tissue Color Modifications

**Figure 2:**
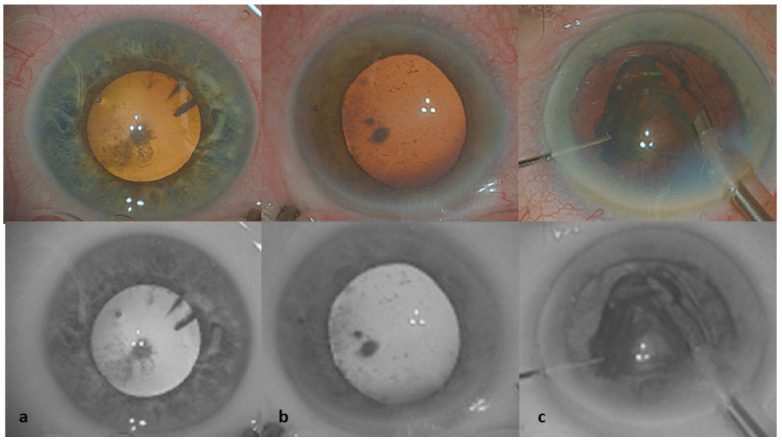
Images obtained during surgery highlighting tissue color modifications with the BW filter. The corresponding natural color images are shown above the black-and-white images. (**a**) Conjunctival vessels tend to disappear with the filter. The red reflex becomes a light, bright-gray reflex. (**b**) Peripheral gerontoxon is less visible with the BW filter. (**c**) The contours of the nucleus were enhanced by the BW filter, but the shades of the colors of the different crystalline layers were attenuated.

The BW filter caused several modifications to natural colors (Figure 2). The red-reflex became a light, bright-gray reflex. The nucleus was light-gray and appeared more transparent, whereas the localized cataract opacities, capsulorhexis coutour, nuclear grooves and fragment contours were dark gray to black in color.

Corneal opacities and peripheral gerontoxon were less visible with this filter. Conjunctival vessels tended to disappear with the filter, and, in cases of bleeding or conjunctival hemorrhagic chemosis, the blood was barely visible and slightly gray in color.

### 3.2. Contrast Measurements and the Surgeons’ Assessments of Tissue Visibility with the BW Filter and Standard Colors during Surgery

The contrast measurements of color and BW filter images are shown in Table 2 for each stage in the surgery, due to the DSD of pixel intensity values > Image contrast in the limbal area was better for standard color images than for images obtained with the BW filter. The surgeons preferred standard color visualization for localizing the limbal area, to make it easier to avoid limbal vessels during incision (Figure 2).

**Figure 3:**
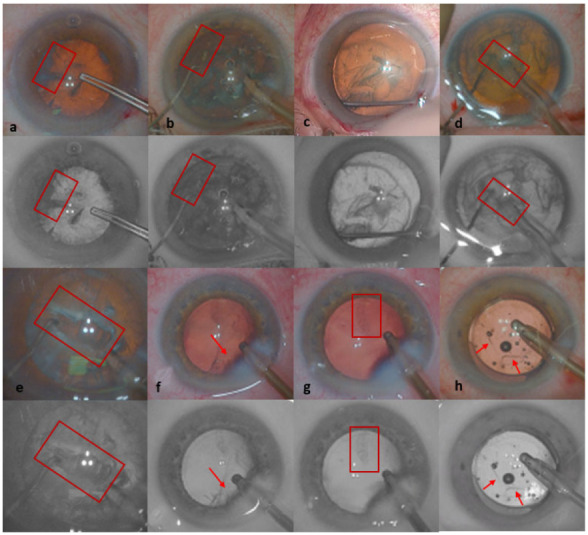
Natural color and BW filter images obtained at the same time points, at various stages in the surgery: Capsulorhexis visibility is enhanced with the BW filter over the anterior subcapsular lens opacities (**a**) and after hydrodissection before phaco-chop in conditions of poor red reflex illumination (**b**). (**c**) Hydrodissection fluid wave perception. (**d**) Enhanced contrast between instruments and the nucleus during phaco-chop. (**e**) The natural colors of the layers of the nucleus provide important information concerning the depth of the groove relative to the posterior capsule. (**f**) Subincisional cortex contrast through corneal edema is improved by the BW filter (arrow). (**g**) Residual cortical debris on the posterior capsule. (**h**) Viscous removal from the anterior chamber (arrows).

The surgeons assessing the images found that the BW filter optimized tissue visibility for capsulorhexis contours, the contrast of the ends of the instruments through opaque nucleus tissue, and subincisional cortex contrast through the corneal edema, facilitating its removal (Figure 3). These findings were concordant with the image contrast measurement values, which were higher for BW filter images during these stages.

The surgeon observers considered hydrodissection fluid wave perception to be better visualized with the BW filter on the basis of video records. However, objective image contrast comparisons were not feasible during this stage of surgery, due to its speed.

Image contrast was greater with the BW filter during nucleus removal, posterior capsular polishing and viscous removal from the anterior chamber. However, the subjective assessments of the surgeons suggested that the BW filter did not make a significant contribution during these stages (Figure 3).

During the nucleus sculpture, contrast did not differ significantly between standard color and BW images. However, the surgeons preferred the standard color visualization for assessments of the depth of the groove (Figure 2).

## 4. Discussion

Standard surgical viewing systems conserve the natural colors of the ocular structures, helping the surgeon to identify subtle anatomic landmarks and ensure safety during surgery. The use of digital technology to apply color filters during surgery can improve contrast, thereby improving the visualization of structures [11,12]. However, such filters should be used with caution because they modify the appearance of tissue colors. Surgeons should take the time to get used to these modifications before using filters in clinical practice.

Unlike the usual optical filters, which block particular wavelengths, the digital BW filter of the Ngenuity system camera works by decreasing the color saturation of the image. Reducing saturation drains the color away, leaving just the grayscale component. A color with 100% saturation contains no white, whereas a color with 0% saturation corresponds to a shade of gray. Decreasing the color saturation implies there being an addition of more whiteness to the color. For example, as saturation is reduced, dark-red colors become a mid-gray, and the light-red color of conjunctival vessels tends towards white, such that these vessels become barely visible. The BW filter converts the red reflex into a light, bright-gray reflex. The tissues take on a gray color, of various scales. Interestingly, the contours of structures are enhanced, becoming dark-gray to black in color, which renders them more visible.

In this series, we report the utility of the BW filter for enhancing contrast and detail discrimination at each stage of cataract surgery, based on the objective contrast measurement data and subjective analyses of video records of surgery by experienced cataract surgeons. The subjective analysis of experienced surgeons was important to confirm the utility of the BW filter images that were obtained, on which visualization contrast is classically enhanced.

Standard color visualization was better than the BW filter for localizing the limbal area, in making it easier to avoid the limbal vessels during incision. Indeed, the limbal vessels were poorly visible with the BW filter, and several cases of chemosis occurred during surgery in our study.

The BW filter improved the visualization of capsulorhexis, fluid wave perception during hydrodissection, visualization of the ends of the instruments through the opaque nucleus during phaco-chop, and subincisional cortex contrast, facilitating removal.

A BW filter could, therefore, be used occasionally, in challenging cases of cataract surgery in which visualization is generally poor, as in cases of corneal edema or opacity, and poor red reflex visualization.

The red reflex, which is produced by the reflection of microscope coaxial light off the retina, does not provide optimal contrast and visibility during cataract surgery [13]. The BW filter has been reported to optimize the visualization of the contours of tissues for internal limiting membrane peeling and to enhance the visualization of the vascular wall, both in ophthalmology and in other fields [14,15,16]. Capsulorhexis visualization is very important in all cataract procedures. However, capsulorhexis is less visible in conditions in which the red reflex is poor, as in patients with hard posterior subcortical cataracts, a loss of corneal transparency, and vitreous hemorrhage. Capsulorhexis visibility is also significantly lower after hydrodissection, but can be enhanced by the use of a BW filter, making it possible to prevent the manipulator hook or phaco-probe from touching the anterior capsule, particularly during use of the phaco-chop technique.

During nucleus sculpture, the grooves were better visualized with standard color visualization, which highlighted the difference in color between the nucleus and subcortical layers, providing the surgeon with important information concerning the depth of the groove relative to the posterior capsule.

Despite the higher contrast of BW filter images during nucleus removal, the surgeons, in their subjective assessments, reported there being no significant advantage of applying BW filters during this stage. Indeed, standard color visualization highlighted the nuances in color at the borders of nuclear fragments and the epinucleus, whereas all these areas appeared to have a similar dark-gray appearance with the BW filter. Furthermore, the application of the BW filter enhanced the contours of the tissues, but attenuated or eliminated the various shades of the natural colors.

It would be interesting to evaluate the utility of BW filters in cases that were complicated by posterior capsular rupture, as a means of enhancing vitreous visualization in the anterior chamber, and the use of other color filters in cataract surgery. Indeed, each color channel has its own optical characteristics, and would be expected to improve the visualization of particular tissues, depending on their colors and compositions [17,18,19].

Recently developed microscopy technologies can provide good standard color visualization of the various structures using sufficiently high contrast qualities, in order for surgery to be performed safely in almost all cases. BW filter use is an additional option that could be used in cases of poor visibility, to enhance the contrast and visibility of capsulorhexis, fluid wave perception during hydrodissection, instrument ends contrast through nucleus during phaco-chop procedures, and subincisional cortex removal.

In conclusion, we describe here the potential of BW filters for use in digital 3D visualization systems, to enhance tissue visualization during cataract surgery. This filter should be used, with caution, in cases of poor visualization, particularly during capsulorhexis, hydrodissection, phaco-chop, and subincisional cortex removal sequences.

## Figures and Tables

**Table 1:** Subjective evaluation and agreement between two surgeon observers assessing for visualization quality between black and white filter and standard colors at each step of cataract surgery.

	Standard Colors	Black and White Filters	Agreement (κ Value)
Limbus identification before incision (*n* = 40)	Better	Worse	1.00
Capsulorhexis (*n* = 40)	Worse	Better	0.86
hydrodissection fluid wave perception (*n* = 40)	Worse	Better	0.74
Nucleus sculpting (*n* = 14)	Better	Worse	0.81
Phaco-chop (*n* = 26)	Worse	Better	0.74
Nucleus removal (*n* = 40)	No difference	No difference	0.70
Cortex removal through subincisional edema (*n* = 40)	Worse	Better	0.67
Posterior capsule polishing (*n* = 40)	No difference	No difference	0.80
Viscous removal (*n* = 40)	No difference	No difference	0.90

**Table 2:** Comparison of contrast (DSD) between color and black and white images during the various steps of cataract surgery.

	Standard Color	Black and White Filters	*p* Value
Limbus area (*n* = 40)	13.06 (1.05)	12.42 (1.37)	0.0006 *
Capsulorhexis (*n* = 40)	10.49 (2.79)	17.04 (5.26)	<0.0001 *
Nucleus grooves during sculpting (*n* = 14)	13.37 (3.27)	13.97 (4.21)	0.22 ^†^
Instrument contrasts through the nucleus during phaco-chop (*n* = 26)	13.82 (2.34)	15.70 (2.77)	<0.0001 ^†^
Nucleus removal (*n* = 40)	11.17 (1.98)	12.06 (2.92)	0.003 *
Cortex removal through subincisional edema (*n* = 40)	13.88 (3.46)	21.34 (5.12)	<0.0001 *
Residual debris on the posterior capsule (*n* = 40)	11.94 (1.83)	14.66 (3.16)	<0.0001 *
Viscous removal (*n* = 40)	15.96 (2.99)	18.08 (4.04)	<0.001 *

* Paired *t*-test. ^†^ Wilcoxon signed-rank test. DSD = Difference in standard deviation of pixel intensity values.

## Data Availability

The data that support the findings of this study are available from the corresponding author, O.S., upon request.
